# Patient experiences with a smartwatch 1L-ECG versus traditional Holter monitoring for ambulatory cardiac rhythm monitoring: a qualitative study

**DOI:** 10.1136/bmjopen-2025-101557

**Published:** 2025-12-07

**Authors:** Evert P M Karregat, Pieter Vooijs, Eric Wierda, Ralf Harskamp, Wim Lucassen, Jelle C L Himmelreich, Eric P Moll van Charante

**Affiliations:** 1 Department of General Practice, Amsterdam UMC Location AMC, Amsterdam, Netherlands; 2 Personalized Medicine, Amsterdam Public Health Research Institute, Amsterdam, Netherlands; 3 Department of Cardiology, Dijklander Hospital, Hoorn, Netherlands; 4 Department of Public and Occupational Health, University of Amsterdam, Amsterdam, Netherlands

**Keywords:** Telemedicine, CARDIOLOGY, eHealth, Primary Health Care, Wearable Devices

## Abstract

**Abstract:**

**Objective:**

To explore patients’ experiences and perspectives on using a direct-to-consumer smartwatch with single-lead electrocardiography (1L-ECG) for ambulatory rhythm diagnostics in primary care.

**Design:**

Qualitative study using semi-structured interviews and thematic analysis.

**Setting:**

Primary care patients referred for ambulatory rhythm monitoring in a diagnostic centre.

**Participant:**

Eighteen adults with paroxysmal palpitations, of whom nine were female patients (50%), aged 32–85 (median 66) years.

**Intervention:**

Participants simultaneously wore a smartwatch with 1L-ECG capability (Withings ScanWatch) and a conventional Holter monitor for 7 days.

**Outcome measures:**

Patient experiences and perceived barriers and facilitators to smartwatch use for rhythm monitoring, reported after the monitoring period.

**Results:**

Patients found the smartwatch more user-friendly and feasible than the Holter monitor. Difficulties included uncertainty about when to initiate recordings, challenges capturing brief episodes and anxiety triggered by automated algorithm outputs. Participants emphasised the importance of accessible healthcare support for interpretation and reassurance.

**Conclusions:**

This study shows that smartwatch-based 1L-ECG monitoring is feasible and acceptable for ambulatory rhythm diagnostics in primary care. Prior to routine implementation, it is crucial to address the identified challenges: particularly anxiety related to algorithm results, uncertainty about when to record and accessible clinician support.

**STRENGTHS AND LIMITATIONS OF THIS STUDY**

- The interview design allowed an in-depth exploration of patients’ experiences, uncovering factors that a (semi-)quantitative design would likely have missed.
- All participants used both a chest-worn Holter device and a smartwatch single-lead ECG simultaneously, enabling direct comparison.
- The limited number of interviewees restricted exploration of potential differences across patient groups.
- Restriction to patients with palpitations as the indication for monitoring may limit generalisability to other patient groups.
- Use of a single smartwatch brand may limit generalisability to other smartwatch ECG devices.

## Introduction

Palpitations are a common complaint in the community.^1^ The physician’s aim during evaluation is to exclude clinically relevant cardiac causes and to detect important conditions, such as atrial fibrillation (AF), which often necessitates prophylactic treatment to mitigate stroke risk.^2 3^ Detection of, as well as ruling out, an arrhythmia requires capturing an electrocardiographic signal during symptoms to obtain a symptom-rhythm correlation.^2^ Unfortunately, patients are often asymptomatic at the time of consultation, necessitating prolonged electrocardiographic (ECG) monitoring to capture paroxysmal symptoms.^4^ Traditionally, this has been accomplished using continuous ambulatory ECG monitoring devices such as Holter monitors.

The compliance with and diagnostic yield of Holter monitors is limited by their relatively short monitoring time and bulky nature with multiple wired electrodes worn on the chest, potentially interfering with daily activities.^5^ Extending the monitoring duration from one to 4 weeks has been shown to almost double the arrhythmia capture rate, emphasising the importance of maximising the monitoring window.^6^

Single-lead (1L-)-ECG devices, which obtain an ECG signal that is similar to that of a lead I on a 12-lead ECG, could overcome the aforementioned limitations. These handheld smartphone-connected 1L-ECG devices often come in convenient forms, such as smartwatches, and are easy to wear and use.^7^ Recent evidence shows that 1L-ECGs are highly accurate in detecting AF, provided they are interpreted by expert readers.^8^ Additionally, monitoring time of these devices is less constrained by battery life. These features make them well-suited for detecting arrhythmias occurring over extended time intervals.

However, before confirming whether a smartwatch 1L-ECG device can serve as an alternative to Holter monitors in symptomatic patients, it is important to explore potential barriers and facilitators experienced by patients. To our knowledge, patient experiences and opinions regarding the use of these devices in an ambulatory setting have not yet been investigated. In this qualitative study, we therefore aimed to explore patients’ experiences and views on the implementation of a direct-to-consumer smartwatch 1L-ECG device for ambulatory rhythm diagnostics.

## Methods

### Study design

**Figure 1:**
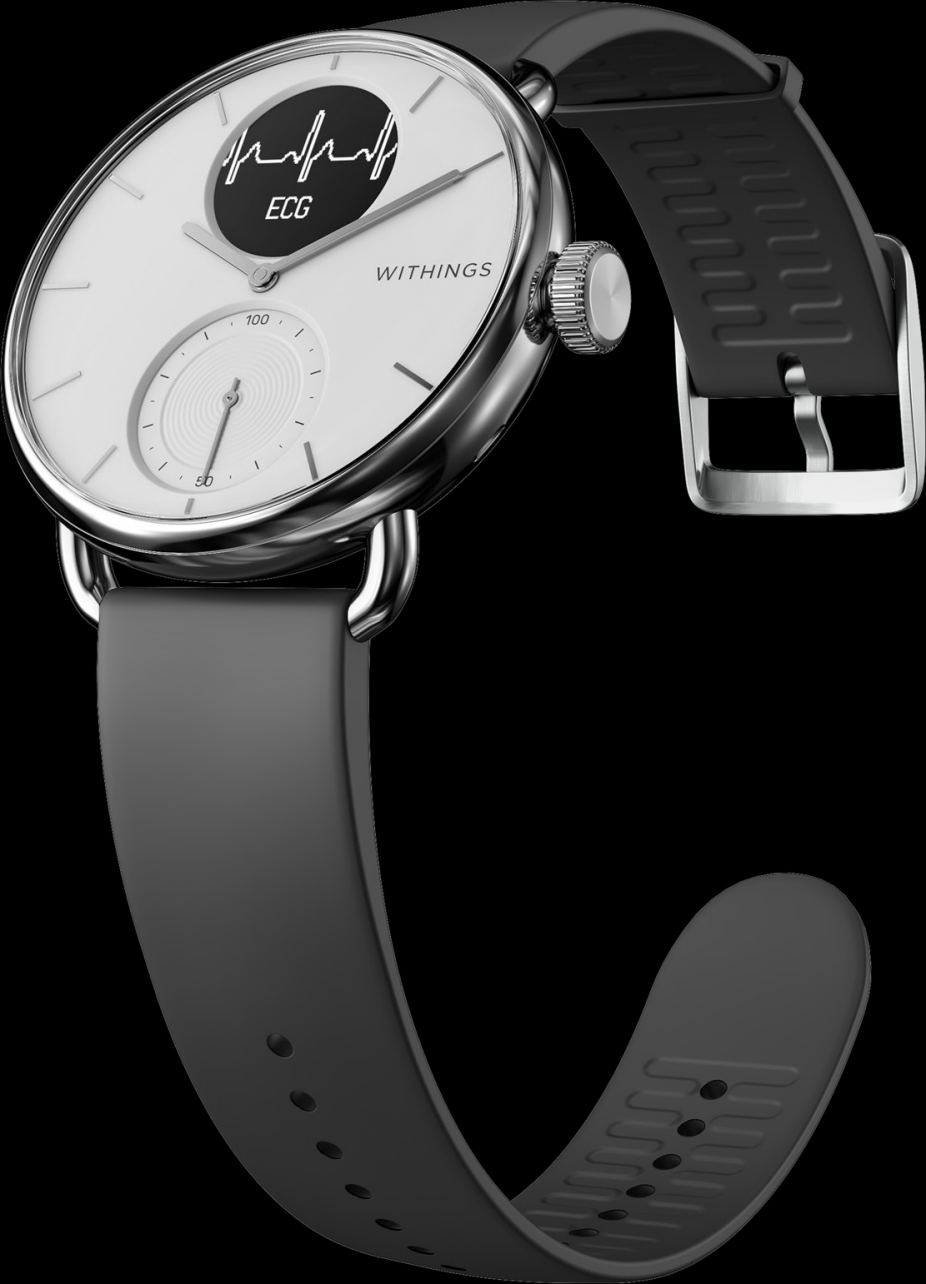
A Withings ScanWatch recording a 1L-ECG. Photo courtesy of Withings Health Solutions.

**Figure 2:**
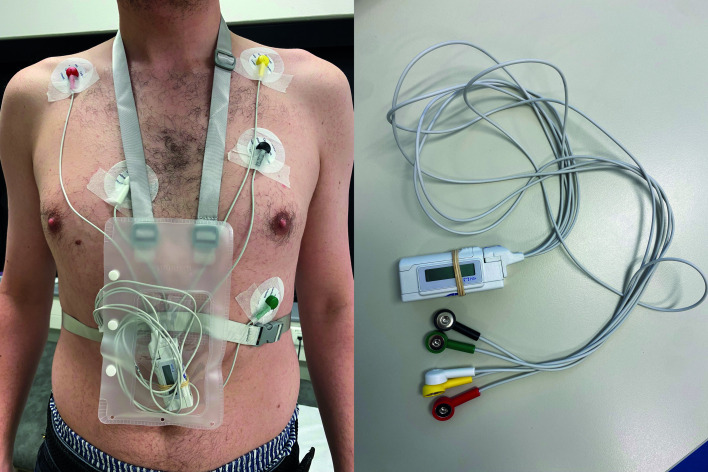
A Welch Allyn H3+Holter monitor (usual care). Photos by Pieter Vooijs.

We conducted a prospective qualitative study among primary care patients referred for Holter monitoring. For this study, we additionally provided participants with a Withings ScanWatch (Withings Health Solutions, Issy-les-Moulineaux, France; see figure 1). This smartwatch provides a 1L-ECG function (the intervention) to be used concurrently with the usual-care Holter monitor: a Welch Allyn H3+ (Welch Allyn Inc., Skaneateles Falls, New York, United States; see figure 2). Further details on cardiac monitoring are provided in online supplemental appendix I. After the monitoring period, we conducted interviews to explore patients’ experiences with the smartwatch and Holter monitor. The study complied with the Declaration of Helsinki principles and received a waiver from the Amsterdam UMC Medical Ethical Board (2022.0335).

### Patient selection

We purposively sampled patients with paroxysmal palpitations who were referred by their general practitioner (GP) to the cardiology outpatient clinic of the Dijklander Hospital, a general non-academic hospital in Hoorn, the Netherlands, for 7-day Holter monitoring. Patients were informed by means of a study information leaflet enclosed with the routine invitation for Holter monitoring. At the outpatient appointment, eligible patients were invited to participate. To maximise diversity, we applied purposive sampling on age group (18–49, 50–74 and ≥75 years), sex and digital literacy, using the mean Digital Health Literacy Instrument (DHLI; 1=low to 4=high) score obtained at the baseline visit.^9^

Patients were ineligible for smartwatch monitoring and subsequent interview if they were unable to operate the smartwatch’s 1L-ECG recorder or to receive adequate instructions for any reason, if they wore a cardiac implantable electronic device, or if they did not own a smartphone compatible with the smartwatch software.

Eligible non-participants were asked to provide limited anonymous demographic data and a brief reason for declining.

**Figure 3:**
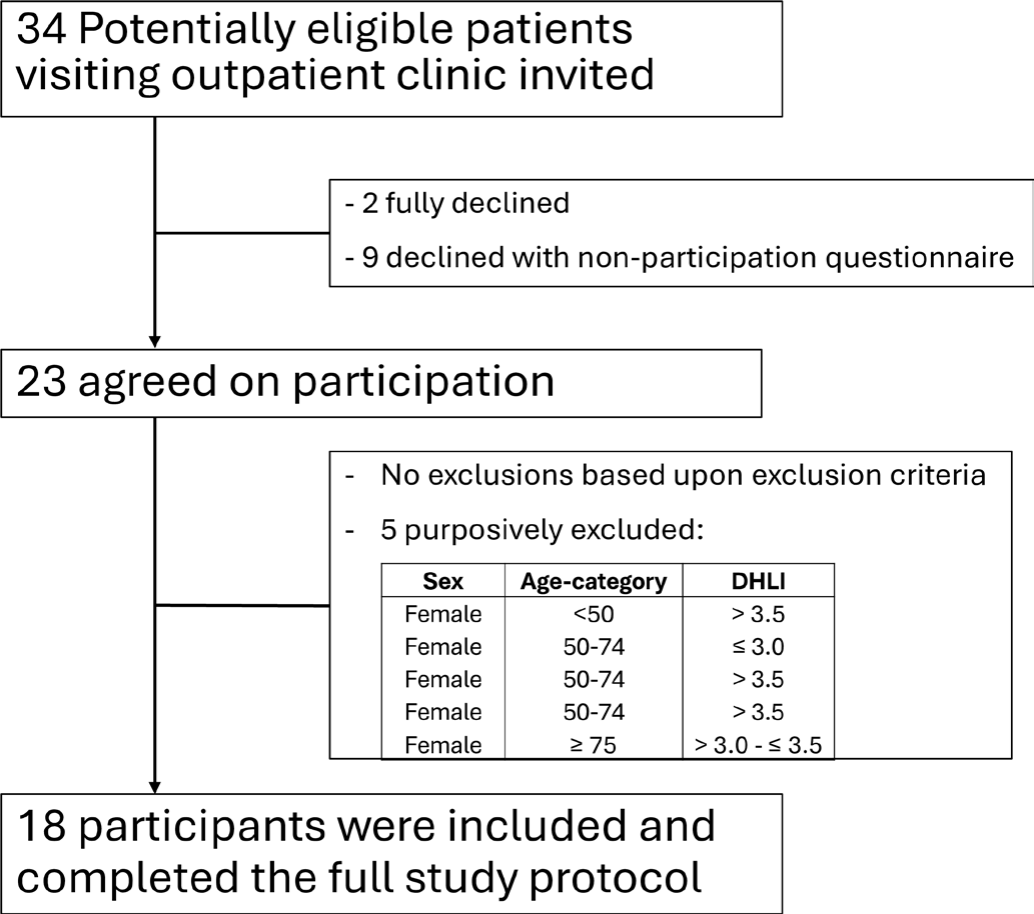
Flow chart of inclusions.

In total, 18 patients participated, completed the full study protocol and were interviewed (see figure 3 for flow chart and table 1 for characteristics). Initially, we invited 34 potentially eligible patients at the outpatient clinic for study participation between January and August 2023. Individuals≥75 years had low presentation rates and often declined participation. Eleven patients declined participation, of which nine agreed to complete the non-participant questionnaire. These nine patients cited concerns about study complexity and smartphone compatibility – even prior to checking compatibility by our researchers (online supplemental appendix II). Twenty-three patients were willing to participate, of which five were purposively excluded due to (a combination of) being women, being of younger age or having higher DHLI, as such participants were already sufficiently represented in our sample (see baseline characteristics of the n=5 excluded patients in the flow chart). Interviews lasted 22–55 min.

**Table 1:** Characteristics of included participants

		Included (n=18)
Age	Range (min-max)	32–85
	Median (IQR)	66 (46–73)
	<50	6
	50–74	8
	≥ 75	4
Sex	Male	9
	Female	9
DHLI	Range (min-max)	2.6–4.0
	≥ 2.5 -≤3.0	6
	> 3.0 -≤3.5	6
	> 3.5	6
Cardiovascular history	None	12 (66.7%)
	Atrial fibrillation	3 (16.7%)
	Other cardiac arrhythmia	0 (0.0%)
	Ischaemic heart disease	1 (5.6%)
	Valvular heart disease	2 (11.1%)
	Type 2 Diabetes	2 (11.1%)
	TIA/cerebrovascular accident	3 (16.7%)
Smoking status	Yes	1 (5.6%)
	Never smoked	9 (50.0%)
	Stopped for more than 1 year	8 (44.4%)
	Stopped less than 1 year	0 (0.0%)
Highest level of education completed	Low: up to pre-vocational secondary education	4 (22.2%)
	Medium: up to secondary vocational education	5 (27.8%)
	High: higher vocational schooling or university	9 (50%)

All items were self-reported with a questionnaire.

DHLI, Digital Health Literacy Instrument.

### Smartwatch

Following inclusion, study staff assisted participants with installing and using the smartwatch and companion smartphone application. To protect privacy, we used dummy data during setup. To facilitate and simplify its use, we customised the smartwatch menu to only display the 1L-ECG recording function. Participants were instructed, on experiencing symptoms, to activate the Holter monitor and then record a 30 s 1L-ECG. After each recording, the smartwatch and its app displayed the results of the automatic algorithm interpretation. If “possible AF” was indicated, the app prompted users to contact a healthcare professional; however, we advised participants to act only when symptoms warranted medical attention. Although not used in this study, other app read-outs (eg, photoplethysmography (PPG)-derived heart rate (with a built-in irregular heart-rate notification), step count and sleep score) remained visible, as these features could not be disabled.

### Data collection

We collected baseline characteristics using a questionnaire covering age, sex, DHLI, cardiovascular disease history, smoking status and education level. Both 1L-ECG recordings and Holter data were retrieved after the 7-day period. As part of usual care, Holter results were subsequently communicated to participants by their GP, typically a few days later. These clinical ECG results were not included in the present analysis.

#### Interviews

After all ECG data had been collected, we conducted semi-structured interviews in a private room at the Dijklander Hospital, using an interview guide with a deductive topic list. The guide was developed using relevant implementation outcomes: acceptability, appropriateness, feasibility and sustainability as described by Peters *et al*.^10^ We adhered to the definitions of these outcomes as described by Peters *et al*: acceptability, being defined as the perception among stakeholders that an intervention is agreeable; appropriateness, as the fit or relevance of an intervention in a particular setting; feasibility, as the extent to which an intervention can be carried out in a particular setting and sustainability, as the extent to which an intervention is maintained or institutionalised in a given setting.^10^ To interpret intentions to use and usage behaviour, we drew on the Unified Theory of Acceptance and Use of Technology model.^11^ To consider social context, we incorporated elements of the Social-Ecological Model.^12^ The guide was deliberately broad to encourage participants to raise additional themes and contribute rich contextual detail.

During interviews, participants compared their experiences using the smartwatch with those using the conventional Holter monitor and were encouraged to speak freely, emphasising no wrong answers were possible.

Two male investigators (EPMK: GP and PhD student and PV: medical master’s student) trained in qualitative research conducted the interviews. Participants and interviewers had met 1 week earlier at inclusion. All interviews were audio-recorded, and field notes captured key observations and contextual factors immediately afterwards. 

### Analysis

Analysis followed an iterative, continuous process. Insights from early interviews were used to refine the interview guide (final version in online supplemental appendix III). Nine participants were re-contacted to clarify emerging questions.

Interviews were transcribed verbatim and anonymised. Instead of transcript return, we performed multiple in-interview member checks. We conducted a thematic analysis according to Braun and Clarke’s six phases.^13^ For the first twelve interviews, two researchers (EPMK and PV) independently completed all phases, resolving discrepancies with a third researcher (EPMvC). For the remaining six interviews, one researcher (EPMK) conducted the analysis, with critical review of each step by EPMvC. Using MAXQDA Plus 2022 (v22.1.1), researchers familiarised themselves with the data, generated and compared initial codes, and collaboratively developed, reviewed and refined themes. Narratives were then drafted to define and name themes, supplemented with illustrative extracts. Recruitment ceased at data saturation as agreed by all three analysts.

Reporting adheres to the Standards for Reporting Qualitative Research.^14^

## Results

We describe our findings from the interviews according to the themes as described in the coding tree (table 2). Regarding the implementation outcome ‘sustainability’ as described in our topic list and interview guide, findings affecting a sustainable implementation were all relatable to one of the other implementation outcomes and were therefore not separately discussed.

**Table 2:** Coding tree

Theme	Subtheme	Subcategory
Acceptability	Burden*	
	Ease of use^†^	
	Duration of monitoring^‡^	
Perceived Appropriateness	Device proficiency^§^	Workflow Privacy Overall preference Reason for participation
	Device reliability^¶^	
	Feedback of device^**^	
	Data handling^††^	
	Performance expectancy^‡‡^	
Feasibility	Overall practical feasibility^§§^	
	Successful recordings^¶¶^	
	Expectations^***^	
Contextual factors	Religion or culture^†††^	
	Influence of location on use^‡‡‡^	
	Influence of important persons on intention to use ^§§§^	

* The experienced burden of wearing and/or using the devices

† The experienced ease of use of the devices

‡ Influence of monitoring-duration on how agreeable participants found using the devices

§ Experienced competence for use by participants

¶ Confidence in the use and performance of the devices by participants

** The experienced appropriateness of the feedback, or lack thereof, that the devices provide

†† Participants’ views on how collected data were handled

‡‡ The expected fit and overall performance of the devices

§§ Extent to which participants felt the devices were usable in daily life for their intended purpose

¶¶ Extent to which participants were able to make successful ECG recordings

*** Participants’ expectations regarding universal usability of the devices (any setting, any user)

††† The experienced influence of religion or culture on the (intended) use of the devices

‡‡‡ The influence of location on the (intended) use of the devices

§§§ The influence of important persons (eg. family, friends, health care professionals) on the intention to use the devices

### Acceptability

#### Burden 

All participants continued to use the smartphone throughout the study without major burdens. They appreciated the convenience of the 1L-ECG device in the form of a (smart)watch and encountered no significant obstacles integrating it into their daily routines.

It’s a bit heavier than my own smartwatch. That takes a minute to get used to, but after that you don’t even notice it anymore. It’s waterproof, so you barely notice you’re wearing it — not even at night, since I always sleep with a watch on. I don’t feel the difference anymore. Other than that, it does what it’s supposed to do: tell the time. Which is pretty handy, too. **P16, male patient, 48 years**

In contrast, participants reported encountering various obstacles with the Holter monitor, including skin irritation or itchiness from the adhesive patches. They also experienced inconvenience and anxiety about inadvertently disconnecting the wires during normal daily activities.

Participants tolerated wearing the smartwatch for extended periods, unlike the Holter monitor, which had more limitations and inconveniences. One participant disconnected the Holter monitor prematurely (P4).

Well, I basically wore the watch all the time. It’s super convenient that you can keep it on while showering, sleeping, and so on […]. If I were to have any symptoms while taking a shower, those patches wouldn’t record anything, but the watch would. Plus, after a while those patches started to get a bit irritating. They make your skin itch, and at some point you really have to replace them. Or the wires get caught on something — that’s just annoying. With the watch, I didn’t have any of that at all. **P15, male patient, 32 years**

#### Ease of use

Overall, participants found operating the smartwatch and activating the 1L-ECG recording to be straightforward. However, some found it challenging to record a 30 s 1L-ECG in certain situations, such as while driving, exercising or in bed during the night. One participant was unable to record any 1L-ECG.

I find it easy… But … when you're not feeling well, with the heart*,* especially at night, then you're not really… able to think: oh, I should sit like this first. **P5, female patient, 58 years**

One participant found the information in the app too extensive for the sake of this study, since it also showed results of the other trackers (eg, pedometer, etc.).

As for using the Holter monitor, a number of participants found it difficult to press the event button. This was often buried beneath their clothes, making it difficult to locate when wanting to press the activation button.

Overall, it went really well. Yes. One major advantage is that you hardly ever need to charge it. That’s definitely a plus. It was also very easy to use - just turn it and hold it. I actually found it easier than the Holter monitor: you have to look for that button each time. **P3, female patient, 65 years**

### Perceived appropriateness

#### Device proficiency 

Participants felt confident using the smartwatch; however, they sometimes experienced uncertainty about whether certain physical sensations warranted recording an ECG. This applied to both the smartwatch and the Holter monitor. As the Holter monitor recorded continuously, regardless of them triggering the device, participants perceived this as an advantage over the smartwatch.

So you have to make a decision yourself: do I make a recording or not? Yes, in one way or another… If it’s just automatically recorded, then you don't have a decision moment. Then you're thinking: am I going to record nonsense? **P3, female patient, 65 years**

None of the participants reported any discomfort or feelings of distress related to the passive screening for irregular heart rhythm using the PPG sensor. In no participant did the passive PPG screening generate a notification of a possible irregular heartbeat.

Well, I have experienced that as perfectly fine. I didn't worry about the watch suddenly giving a notification. I didn't really think about it, just wore the watch as usual. **P11, male patient, 44 years***.*

#### Device reliability

Participants expressed various degrees of confidence in the smartwatch as a reliable diagnostic tool for cardiac rhythm assessment. While some were (strongly) convinced of its reliability in diagnosing cardiac arrhythmias, others expressed more ambivalent views. The latter group mainly doubted its reliability because the smartwatch was provided in a study setting, and because they perceived the Holter monitor as more comprehensive.

(on why participant thinks the Holter monitor is better:) because the watch looks more like a commercial product and the Holter monitor more like medical equipment. Also because it simply measures at multiple points. Also because it makes a continuous recording instead of a snapshot. So, it is all about perception, I think […] The watch also indicates itself that it sometimes doesn't measure well or couldn't make a recording. Then you're less certain that it did it correctly. **P9, male patient, 39 years**

Participants indicated that they would not only support but also prefer the use of the smartwatch as an ambulatory rhythm diagnostic when provided with more evidence on its reliability.

If those watches pass the trial and give good results compared with that recorder, then that would actually be much easier. **P18, male patient, 79 years**

#### Feedback

Some participants appreciated receiving feedback confirming that an event-ECG was recorded successfully, a feature which the Holter monitor lacked.

(on the Holter monitor): It’s a button you have to press. Then you think: did I do it right? It doesn't indicate that it’s recording. I would appreciate that in such a device, that you're sure it’s recording. **P7, female patient, 47 years**

As for the automatic algorithm interpretation in the app, participants felt reassured when it provided a normal result. However, in two cases, the algorithm detected a possible arrhythmia, prompting participants to contact their physician. One participant (P8, contacted the out-of-hours GP service on receiving this notification. However, the other (P1) experienced anxiety but followed our instructions to act on the physical complaints only.

[I felt anxiety] to some extent, but … I just know what causes it. It's just due to stress. Then the heart races. I don't worry extra about that. … Yes, of course [I hesitated to call the healthcare provider] … things really need to go wrong before you actually contact your healthcare provider. **P1, male patient, 71 years**

Additionally, some participants encountered unreadable results from the smartwatch’s automatic interpretation, leading to feelings of insecurity.

The smartphone app also displayed results from the PPG sensor, pedometer and sleep-tracker. Although these were beyond the scope of this study, our participants mostly appreciated this additional feedback in the smartphone app, finding it positively motivating and facilitating sustained use of the smartwatch.

I also really liked that it tracks your sleep — that you can see what’s happening there, including your heart rate and so on. That’s something I really appreciate about the smartwatch. **P10, female patient, 32 years**

#### Data handling

Participants found using their personal smartphones convenient, as all data were readily accessible in the app. While no major obstacles arose during data collection, they recommended expediting the delivery of investigation results to their GP, mainly for those having received abnormal algorithm results.

(on assessment of the ECG recordings only after a week): Well, I don't like that… Because then you have a week where you're wondering if it was something serious or not. […] But yes, that also applies to the Holter monitor of course. **P2, female patient, 66 years**

(on collection of ECG data): I think that could definitely be made easier. If this app were to be used in practice, I think there should be a way to share the recordings with your healthcare provider — maybe through your patient portal or something like that.[…] Either that you can send it yourself, or that — if you’re in a care process — you could give consent for data collection during a specific period, for example. **P11, male patient, 44 years**

#### Privacy

No major privacy concerns were raised by our participants. Some mentioned being accustomed to their personal data being stored in various places. This attitude may be attributed or reinforced by the nature of the data (ECG data), which may appear abstract or impersonal to individuals without a medical background.

It’s just heart rate data. If someone wants to see that, that’s fine. […] If you look at social media, I think there’s probably more known about someone there than through this. **P10, female patient, 32 years**

Additionally, participants appreciated our clear explanation of the study procedures and the use of dummy data in the smartphone app, contributing to a sense of security. They also expressed confidence in the hospital supporting the study, trusting them to safeguard their data.

At first, I hesitated: should I do it, should I not? […] Then I think: it’s coming from the Dijklander hospital, it will all be fine. **P6, male patient, 67 years**

### Feasibility

Most participants found the use of a smartwatch with connected smartphone app feasible as cardiac rhythm diagnostic. They valued receiving thorough instructions and an additional instruction manual. They recommended adding an instruction video to watch at home. Additional low-threshold contact possibilities were suggested in case of problems such as a chat function.

I also liked having the instructions on paper, so I could read them again later. Yes, I really appreciated that manual. I received a lot of information during the first visit, so it was nice to be able to go over it again afterwards. **P13, female patient, 79 years**

An important obstacle for the successful recording of a 1L-ECG during symptoms was that some symptoms lasted too short a time to timely activate a 1L-ECG recording on the watch (eg, skipping heart beats).

Yeah, I think it’s super easy it’s also user-friendly. But my complaints; it doesn't quite fit. It skips for me and by the time I press it, that skipping is already gone. I felt like it doesn't register that restless feeling that I have. **P7, female patient, 47 years**

### Contextual factors

Participants encountered no obstacles recording a 1L-ECG with the smartwatch in a variety of social settings. However, triggering the event button of the Holter monitor could lead to social awkwardness, primarily because the device, worn underneath their clothes, required lifting to activate.

I'm not easily embarrassed, but I can also imagine some people thinking: just forget about it… Especially as a woman, it’s a bit less fun to pull up your shirt. With a watch, you don't have that problem, of course. **P16, male patient, 48 years**

Furthermore, the interviews revealed that, for some participants, the opinions of friends and family influenced their motivation to use the smartwatch or Holter monitor. All participants valued the opinion of their healthcare provider regarding the use of either device and indicated they would follow professional advice.

Well, you tend to rely on the expert, don’t you? If they say the Holter monitor is actually more reliable than the watch, then I’d go with that. I’d follow their advice — who am I to say otherwise? **P2, female patient, 66 years**

## Discussion

This qualitative study showed a smartwatch 1L-ECG device is feasible for ambulatory rhythm diagnostics from a patient’s perspective. Participants found the smartwatch user-friendly and less burdensome compared with a Holter monitor. However, some barriers for implementation were identified. Notably, some symptoms are too short-lasting to capture with a self-triggered smartwatch 1L-ECG, and patients may doubt which symptoms justify an ECG recording. Moreover, we observed that feedback from the automatic algorithm can promote a feeling of safety when normal but also cause anxiety when abnormal. Remote patient monitoring was not essential according to participants; however, it could facilitate extended usage and enhance reassurance when algorithm findings are abnormal.

### Comparison with existing literature

To our knowledge, this is the first qualitative study investigating patient experiences using a smartwatch 1L-ECG in an ambulatory setting for cardiac rhythm monitoring.

Previously, Ding *et al* studied the experiences of post-stroke patients using a smartwatch with a PPG sensor to detect an irregular pulse indicative of AF.^15^ A difference with our study was that their participants did not have to actively record an ECG. As in our work, participants in Ding *et al* found a smartwatch easy to use and more comfortable than a Holter monitor. The benefits of the smartwatch included direct feedback and a sense of security by participants. They also appreciated the accessibility of their research team, similar to our findings. However, their participants struggled with the large number of features on the smartwatch’s menu, hindering usability. Participants in our study found the smartwatch’s menu user-friendly, likely because our research team disabled all functionalities except the 1L-ECG function. Lastly, in the study of Ding *et al* patients needed to recharge the smartwatch frequently, causing inconvenience and a feeling of insecurity due to interrupted monitoring. In our study, the smartwatch battery did not need recharging during the 1- week monitoring period.

Other studies investigating user experiences with a smartwatch 1L-ECG device have been done; however, in our opinion, these are difficult to compare to our study. Shih *et al* interviewed early consumers of the Apple Watch with ECG feature.^16^ This was a relatively young population of ‘worried-well’ instigating self-screening with their Apple watch. Therefore, this was an asymptomatic, relatively young population using the 1L-ECG smartwatch device not initiated or supervised by a physician. The focus in their interviews, and thereby their results, was therefore not comparable to our setting and population. Koshy *et al* also investigated patient experiences when simultaneously using a chest-worn device and smartwatch.^17^ However, these were hospitalised patients in a tertiary hospital, of whom almost half did not own a smartphone. Therefore, their population was not similar to an ambulatory primary care cohort with a requirement for smartphone ownership as in our study. Lastly, Koshy *et al* used a questionnaire instead of a qualitative method to further explore patients’ experiences and views.

We found that the feedback provided by the smartwatch could lead to different behaviours. Some feedback was considered a positive motivation for sustained use of the smartwatch, which is consistent with the findings of Seto *et al* who piloted a telemonitoring system among heart failure patients.^18^ Furthermore, when the 1L-ECG algorithm returned a normal result, participants experienced a sense of reassurance. This aligns with findings by Rosman *et al*, who investigated the psychological response of patients with known AF to rhythm results of a wearable.^19^

Contrarily, an abnormal algorithm result from a 1L-ECG recording could trigger anxiety among participants. This anxiety response was similarly reported by Rosman *et al*, who found that abnormal rhythm results of a wearable could induce distress and lead patients to seek medical attention.^19^ These findings highlight the need for healthcare professionals to consider anxiety as a potential drawback of home monitoring when devices don’t conceal automatic algorithm results from patients.

We found that a thorough explanation, along with the support of a major healthcare institution, helps facilitate participants’ trust regarding their privacy. This is consistent with Ginsberg *et al* who, in a recent review, address key issues for wearable digital health technologies.^20^ A new factor we found is that patients can develop a somewhat indifferent attitude towards their privacy, which may be strengthened by the abstract nature of ECG data.

### Strengths and limitations

To our knowledge, this is the first qualitative study investigating the use of a smartwatch 1L-ECG among primary care patients referred for rhythm monitoring in an ambulatory setting. Moreover, since our participants simultaneously received a Holter monitor, they were able to make an honest comparison between the two.

Some limitations also deserve mentioning. First, regarding our population. We only included participants with palpitations, whereas patients may also receive a Holter monitor for other symptoms. Additionally, it proved difficult to recruit people aged ≥75 years. It is possible that older patients may feel insufficiently capable of participating in research involving digital innovations, as increasing age is a known factor negatively influencing the use of digital technology.^21^ Second, the simultaneous use of both devices could have affected the overall evaluation of both modalities. Certain functionalities that one device has, but the other lacks, can become more apparent. Our ethical board did not permit us to evaluate their subsequent use, since this could have negatively impacted the quality of usual care. Third, some results may be influenced by the specific brand and type of smartwatch used in this study. We selected this device for its user-friendly interface, long battery life and compliance with EU data and privacy regulations. This limitation also applies to the Holter monitor used at the Dijklander Hospital: other Holter monitors and patches may be less burdensome, though a recent study found participants also disliked the 3-day monitoring time of a single-lead patch.^22^

### Implications for clinical practice

Participants in this study found the smartwatch easy to use with minimal burden; however, several implementation barriers were identified. First, while participants had no concerns about the current assessment period of just over a week before receiving results, an extended monitoring period would increase the need for a remote patient monitoring system to enable real-time assessment by healthcare professionals. Such a system could also promote patients’ sense of safety, particularly for those receiving abnormal automatic algorithm results. Alternatively, disabling the display of these algorithm-generated results for participants when used in professional medical settings could help mitigate anxiety while maintaining diagnostic integrity. Second, clear instructions and adequate instruction materials, together with low-threshold communication options, are recommended. Lastly, short-lasting symptoms (such as extrasystoles) could not be recorded with the smartwatch 1L-ECG. This limitation can be addressed by selecting only patients with longer-lasting symptoms for rhythm monitoring with a smartwatch 1L-ECG. Another suggestion was to equip the smartwatch with a continuous monitoring function.

### Recommendations for future research

Further research should explore healthcare professionals’ experiences with 1L-ECG smartwatches, as they will be the first to handle these devices. Studies are also needed to optimise data management without overloading the healthcare system. Additionally, the impact of displaying vs blinding algorithm results on patient anxiety requires investigation.

To our knowledge, the diagnostic accuracy and yield of 1L-ECG smartwatches for ambulatory rhythm diagnostics have never been compared with a reference standard. Therefore, further research comparing these two modalities is warranted to determine the optimal role of 1L-ECGs in clinical practice.^23^ This could also enhance patients’ trust in the smartwatches’ diagnostic reliability.

## Conclusion

Our study demonstrates that 1L-ECG smartwatches are a feasible and user-friendly alternative to Holter monitors for 7-day monitoring. However, from a patient perspective, concerns remain about justifying recording triggers based on physical sensations and capturing brief symptoms using a self-triggered smartwatch. Addressing potential barriers, like anxiety from abnormal algorithm results and a desire for low-threshold contact options with healthcare providers, is crucial before widespread implementation.

## Supplementary material

10.1136/bmjopen-2025-101557 online supplemental file 1

## Data Availability

Data are available upon reasonable request.
