# Correction for ‘patient experiences with a smartwatch 1L-ECG versus traditional holter monitoring for ambulatory cardiac rhythm monitoring: a qualitative study’

**DOI:** 10.1136/bmjopen-2025-101557corr1

**Published:** 2026-02-05

Karregat EPM, Vooijs P, Wierda E, *et al* Patient experiences with a smartwatch 1L-ECG vs traditional Holter monitoring for ambulatory cardiac rhythm monitoring: a qualitative study. *BMJ Open* 2025;0:e101557. doi:10.1136/bmjopen-2025–1 01 557

This article has been corrected since it was published online. Unfortunately, the alignment of table 1 was not displayed correctly, whereby the numbers presented in the second column did not correspond to the correct corresponding characteristics presented in the first column. With this correction, this issue has been resolved.

Here below, you find the correct table:

**Table 1:** Characteristics of included participants

		Included (n=18)
**Age**	**Range (min-max**)	32–85
	**Median (IQR**)	66 (46–73)
	**<50**	6
	**50–74**	8
	**≥ 75**	4
**Sex**	**Male**	9
	**Female**	9
DHLI*	**Range (min-max**)	2.6–4.0
	**≥ 2.5 -≤3.0**	6
	**> 3.0 -≤3.5**	6
	**> 3.5**	6
**Cardiovascular history**	**None**	12 (66.7%)
	**Atrial fibrillation**	3 (16.7%)
	**Other cardiac arrhythmia**	0 (0.0%)
	**Ischaemic heart disease**	1 (5.6%)
	**Valvular heart disease**	2 (11.1%)
	**Type 2 Diabetes**	2 (11.1%)
	**TIA/cerebrovascular accident**	3 (16.7%)
**Smoking status**	**Yes**	1 (5.6%)
	**Never smoked**	9 (50.0%)
	**Stopped for more than 1 year**	8 (44.4%)
	**Stopped less than 1 year**	0 (0.0%)
**Highest level of education completed**	**Low: up to pre-vocational secondary education**	4 (22.2%)
	**Medium: up to secondary vocational education**	5 (27.8%)
	**High: higher vocational schooling or university**	9 (50%)

All items were self-reported with a questionnaire.

* Digital Health Literacy Instrument.

