# Supplementary material for: Patient experiences with a smartwatch 1L-ECG versus traditional Holter monitoring for ambulatory cardiac rhythm monitoring: a qualitative study
Source: BMJ Open. 2025 Dec 7;15(12):e101557. doi: 10.1136/bmjopen-2025-101557 (PMC12684167; doi:10.1136/bmjopen-2025-101557)
Supplement: online supplemental file 1 [file bmjopen-15-12-s001.docx]

**Supplementary material to Karregat et al., “Patient experiences with smartwatch versus traditional Holter monitoring for ambulatory cardiac rhythm monitoring – a qualitative study”**

**Appendix I: Supplementary methods**

Smartwatch 1L-ECG

We used a Withings ScanWatch (Withings Health Solutions, Issy-les-Moulineaux, France). This smartwatch is able to record a 30-second 1L-ECG, which can immediately be assessed on a paired device (e.g. smartphone) using the Health Mate app (Withings Health Solutions, Issy-les-Moulineaux, France). Users can activate the 1L-ECG function using the crown stone on the smartwatch, after which the recording starts by having the contralateral hand hold the smartwatch’s housing (see Appendix II). This smartwatch is also equipped with photoplethysmography (PPG) technology, which can monitor the wearer’s heart rate and passively screen for an (asymptomatic) irregular pulse. To diagnose a cardiac arrhythmia, an (1L-)ECG recording is still needed. Other functions of the ScanWatch, such as oxygen saturation, a pedometer and a sleep score, were not used for this study. However, participants could still view these parameters in the app, as it was not possible to completely shield these features.

The recorded 1L-ECGs are automatically assessed by an algorithm. Three results can be displayed based on the algorithm's assessment: ‘normal sinus rhythm’, ‘possible AF’ and ‘inconclusive’.

Usual care Holter monitor

A Welch Allyn H3+ was used for Holter monitoring (Welch Allyn Inc., Skaneateles Falls, New York, United States; see Appendix III). Unlike the smartwatch, this Holter monitor continuously recorded and stored 24/7 ECG data. To establish a symptom-rhythm correlation, patients were also instructed, as part of usual care, to trigger an “event-button” on the Holter monitor, and/or to write down their symptoms and the corresponding time in a diary.

Monitoring procedures

After consent for inclusion, we instructed patients in installing and using the smartwatch and its compatible app on their personal smartphone. To protect participants’ privacy we used dummy data during app setup. Additionally, to facilitate and simplify its use, we customized the smartwatch menu to only display the 1L-ECG recording function.

After setup, we instructed patients on recording a 1L-ECG. The smartwatch’s crown stone served as a hot-key; pressing it for 3 seconds initiated a 1L-ECG recording. In case of symptoms, patients were advised to press the Holter monitor button first, followed by the crown stone on the smartwatch to obtain an immediate 1L-ECG recording. We also instructed participants to record a 1L-ECG in case the PPG sensor notified them of an asymptomatic irregular heart beat (this did not occur in any of our participants’ monitoring period). The Health Mate app prompted participants with "possible AF" to contact their healthcare provider. However, we instructed participants not to contact us or their GP unless their symptoms warranted professional medical attention. Lastly, participants received a pocket card with usage instructions and contact information for the research team.

1L-ECG data collection

After the 7-day monitoring period we obtained the 1L-ECG recordings by securely sending these from the patients’ phones to us investigators. We subsequently removed all data from their personal smartphones and deleted the dummy Health Mate and email accounts. The recorded 1L-ECGs were not used for diagnostic or treatment purposes, and were not part of the current analysis.

**Appendix II: Reasons for declining participation**

| **n** | **Age-category** | **Sex** | **DHLI-category** | **Reason not to participate** |
| --- | --- | --- | --- | --- |
| 1 | 18-49 | Female | ≥2.5 - ≤3.0 | "Have already participated in other studies before." |
| 2 | 50-74 | Female | > 3.5 | "Due to a poorly functioning mobile phone, sorry the battery is "quickly" empty several times a day!" |
| 3 | ≥75 | Female | > 3.5 | "Sleep at night with braces on both wrists. Sleep with apnoea machine, so can't have this too." |
| 4 | ≥75 | Male | ≥2.5 - ≤3.0 | "I don't have a smartphone on which I can install apps." |
| 5 | ≥75 | Male | >3.0 - ≤3.5 | "Not having a properly functioning smartphone, and also don't want to spend too much time dealing with it." |
| 6 | 50-74 | Female | >3.0 - ≤3.5 | "Don't want to have anything else in addition to those patches during the holidays.' |
| 7 | ≥75 | Male | >3.0 - ≤3.5 | "Too complicated." |
| 8 | ≥75 | Male | >3.0 - ≤3.5 | "Too much of a hassle." |
| 9 | ≥75 | Female | N.A. | "It made me nervous. With the computer and everything. […] I feel restless." |

N.A.: Not available.

**Appendix III: Interview Guide for Semi-Structured Interview Regarding WATCH-ECG study**

*Prior to the interview: check if the Informed Consent form has been signed by the participant and researcher (this should always be the case if the watch has been used).*

**Interview Guide**

1. Introduction to the interview

2. Topic list

3. Detailed interview guide

4. Member check

5. Conclusion

**1. Introduction to the interview**

My name is Pieter Vooijs / Evert Karregat, we also met during the distribution of the watch. I am (a master's student in Medicine at the Amsterdam UMC and I'm doing my research internship / a general practitioner and a PhD candidate at the Amsterdam UMC).

As you know, we are interested in the role that handheld devices for recording heart rhythms, such as a smartwatch, can play in heart rhythm diagnostics. Over the past week, you have worn and used a smartwatch simultaneously with the hospital's heart rhythm monitor. With this interview, we want to explore your experiences with this.

The findings from this conversation will only be used for research purposes and will not be shared with your treating physician. All data and information will be treated confidentially. The audio recordings of the interview will be deleted once the analysis is complete.

I would like to emphasize that this is about your own story and experiences. There are no right or wrong answers, so please feel free to speak openly. Occasionally, I will ask questions for clarification or move on to the next topic. This conversation will likely last about three quarters of an hour (up to a maximum of 1 hour).

For completeness, I would like to remind you once again that this interview will be recorded. If you need a break or if there's something you'd rather not have recorded, we can pause the recorder. Are you still okay with that?

Do you have any questions or comments at this time? If not, I would like to start the recording now.

*➔ Start recording*

*Reconfirming informed consent during recording*

**2. Topic list**

• Patient background e-health and m-health

• Overall impression of the watch

• Acceptability of the watch

• Appropriateness

• Social influence

• Data management and privacy

• Feasibility

• Sustainability

• Member check

• Conclusion

**3. Detailed interview guide**

3.1 Patient background e-health

- Do you already use health apps on your phone?
- Do you use the internet/apps for health-related matters? Such as: electronic health records, lab results, making appointments online with the doctor.
- If yes: what are your experiences with this? If no: why do you not use this?
- SEM: Access (financial) to resources to participate in e-health Do the costs of such e-health applications still play a role in whether or not you use these apps?
- What made you decide to participate in this research?

*Member check / summary*

3.2 Overall impression of the watch

How did it go? (Open question, initial impression of the week with the smartwatch.)

Did you experience any complaints in the past week for which you recorded a heart rhythm with the watch?

- if yes: did the watch give a notification that a possible heart rhythm disorder was detected in the recorded heart rhythm?
- What was your reaction to this result? (e.g., anxiety; confirmation of suspicion; reassurance; other?)

The watch is also equipped with a sensor capable of detecting an irregular heartbeat. Did the watch give a notification during the past week?

• if yes: what was your reaction to this? (e.g., anxiety; feeling of safety)

• if no: how do you think you would react to A) a reassuring result; and B) an abnormal result? What does this mean for safety? Is that safety also present when the event recorder was not present? What do you miss without the watch???

*Member check / summary*

3.3 Acceptability of the watch

How easy is it to wear and use the smartwatch in daily life compared to the event recorder? (consider perceived difficulty/workload compared to event recorder with patches)

• is this perceived as a burden, and if so: how exactly?

• Is the watch easy to use and operate? (also compared to the event recorder)

• Is the app easy to use? (also compared to the event recorder)

• During the complaints, were you able to make a recording on the watch? Or did you often get "unclear" results, requiring a new measurement?

Form: prefer smartwatch or other form (consider mydiagnostick or smartphone paired but prefer for example KardiaMobile)

• would a smartwatch be suitable for everyone?

What were your experiences with using this smartwatch?

• was the smartwatch more easily integrated into your daily life?

• Was it possible to make a heart rhythm recording in every situation - when necessary (e.g., you were on a train or at the movies)

How did you experience using the event recorder?

• was the event recorder easily integrated into your daily life?

• Was it easy to use in different social situations (e.g., train or movies)

• Is the 'visibility' of the ER (or the watch) perceived as a hindrance / annoyance by participants?

How did you experience the explanation and instructions during distribution?

• was this sufficient?

• can something be improved here?

*Member check / summary*

3.4 Perceived Appropriateness of the watch

Does a smartwatch with a 1L-ECG function seem like a useful diagnostic tool to you?

Do you trust a heart rhythm recording made with the watch to detect or exclude heart rhythm disorders? (and why yes/no)

Where does that trust come from exactly?? How can we create that trust if we were to implement another mHealth tool?

The smartwatch has some limitations compared to the event recorder, namely that you have to decide for yourself to make an ECG recording and the fact that it does not record continuously, when taking everything into account: how do you see the place of the smartwatch for diagnostics of heart rhythm disorders compared to the (current) event recorder?

• does the duration of monitoring / wearing still influence this opinion?

*Member check / summary*

3.5 Social factors

Were there social barriers in using the smartwatch?

Were there social factors that were encouraging in using the smartwatch?

Do you come from a certain culture or religion that - due to certain core values - has encouraged or hindered you in the use of both the smartwatch and the event recorder?

• If yes: how did you experience the role of this on your eventual use of the smartwatch?

• How did you experience the reactions of other people in your environment to the fact that you wore and used this watch? (e.g., your partner?)

• SEM: Do you mind making a recording in the presence of others?

• Would they recommend you to use this?

• Would the opinion of your friends/family influence your motivation to use it?

• Would you recommend it to other people with similar complaints?

*Member check / summary*

3.6 Data Management and Privacy

Do you have concerns about your privacy when using such a (commercially available) watch?

• Why yes/no (due to use of dummy data? / type of data?)

• Does the way the heart rhythm recordings are shared with your healthcare provider still affect this?

• How do you think the recorded heart rhythm should be shared with your healthcare providers? (e.g., automatic upload; or could this be done via email?)

• Would you prefer the smartwatch to be linked/not linked to your own smartphone?

• When would you have privacy concerns?

• Have you ever had privacy issues with other digital data?

• if yes: why (not) now? (medical setting? otherwise?)

How do you feel about the fact that the recorded heart rhythms are reviewed by a healthcare professional after 1 week, and not immediately?

• Would an instant connection with the healthcare provider for faster assessment and results be your preference?

o If yes: why?

Privacy: does it matter if the ECG or the personal data are exposed to others?

There are few privacy concerns. Is that also the case when they would be asked to email the recordings to their healthcare provider using their private email address (e.g., Gmail, Hotmail)? And where does that trust come from exactly? Trust in regulations? Or not caring if someone else sees their data?

*Member check / summary*

3.7 Feasibility

Do you find it feasible - based on your experiences over the past week and regardless of your preference - to use a smartwatch for heart rhythm diagnostics?

Were there practical barriers?

Were there practical factors that were encouraging you to use the smartwatch?

• Did you find the use of the smartwatch and the app so easy that it could be used for every complaint according to your opinion?

• What do you think could be improved?

Do you think your experience would be the same whenever you were not simultaneously wearing an event recorder? (usability / feeling of safety / etc.)

*Member check*

3.8 Sustainability

How did you feel about constantly wearing a watch that could give a notification at any moment that the heart rate was abnormal?

• How was this for your partner? Did it give a sense of security or rather anxiety?

If the watch's battery dies, would it be a problem for you to carry the charger and charge it yourself?

What would you think if your healthcare provider (general practitioner or cardiologist) wanted to give you this watch OR the event recorder for a longer period?

• Does the duration in which the device must be worn still affect this opinion?

• What other factors play a role in agreeing or disagreeing with using the watch or the event recorder for a longer period?

• Does your healthcare provider (General practitioner or cardiologist) play a role in your motivation to use the smartwatch or event recorder?

Would you consider buying a smartwatch with such heart rhythm and ECG sensors yourself?

o If yes: why? (feeling of security?)

o If no: why not?

*Member check / summary*

**4. Member Check**

• The interviewer provides a summary of what he/she heard from the patient.

• Actively ask if the interviewer understood it correctly.

• Do you have any further additions?

• Is the summary correct?

• Do you have any specific topics where you would like to provide further elaboration?

**5. Conclusion**

• Do you have any further additions and/or questions?

• Thank you very much for your cooperation and openness.

• You can always contact us for questions and comments.

• Hand over the voucher for participation in the research.
