# Supplementary material for: Patient experiences with a smartwatch 1L-ECG versus traditional Holter monitoring for ambulatory cardiac rhythm monitoring: a qualitative study
Source: BMJ Open. 2025 Dec 7;15(12):e101557. doi: 10.1136/bmjopen-2025-101557 (PMC12684167; doi:10.1136/bmjopen-2025-101557)
Supplement: Supplementary file 2 [file bmjopen-2025-101557.reviewer_comments.pdf]

PEER REVIEW HISTORY

BMJ Open publishes all reviews undertaken for accepted manuscripts. Reviewers are asked to complete a checklist review form and are provided with free text boxes to elaborate on their assessment. These free text comments are reproduced below.

ARTICLE DETAILS

Title (Provisional)

Patient experiences with a smartwatch 1L-ECG versus traditional Holter monitoring for ambulatory cardiac rhythm monitoring – a qualitative study

Authors

Karregat, Evert P.M.; Vooijs, Pieter; Wierda, Eric; Harskamp, Ralf; Lucassen, Wim; Himmelreich, Jelle C.L.; Moll van Charante, Eric P

VERSION 1 - REVIEW

**Reviewer**	**1**
**Name**	**Wennerholm, Carina**
**Affiliation**	**Linkopings universitet Institutionen for medicin och halsa**
**Date**	**15-Apr-2025**
**COI**	**None**

Based on my assessmen, the results and their analysis require further clarification before the manuscript can be considered for publication.

2. **Is the abstract accurate, balanced, and complete?**

- It should be clear that a thematic analysis has been conducted.

4. **Are the methods described sufficiently to allow the study to be repeated?**

- Isn't this a prospective qualitative study with a deductive approach?
- The theoretical framework of the Unified Theory of Acceptance and Use of Technology model, focusing on implementation outcomes? Deductive analysis? This needs to be made clear
- Missing a brief summary of the main content of the interview guide and then a reference, better than including the entire interview guide as an appendix.
- Analysis: A more detailed description of the different steps of the analysis is desired.

5. **Are research ethics (e.g., participant consent, ethics approval) addressed appropriately?**

- Is there an ethics approval number?

10. **Are the results presented clearly?**

- As an overview and aid for the reader, it is desirable to have a table of the themes and sub-themes of the results at the very beginning of the results section.
- I think the first paragraph of the results belongs in the Methods section.
- The thematic analysis seems a bit strange to me. Are the themes in the results abstracted? It seems based on the "topic list" in the interview guide, **Acceptability** and **Perceived Appropriateness**? How was the "topic list" developed? Based on the theoretical framework of the Unified Theory of Acceptance and Use of Technology model?
- There also needs to be an explanatory text under each Theme.

**Reviewer**	**2**
**Name**	**Lobban, Trudie**
**Affiliation**	**Arrhythmia Alliance**
**Date**	**10-Jun-2025**
**COI**	**None**

---

need to engage with patient organisations to reach a far larger group of patients rather than only 18. Organisations that specialise in heart rhythm disorders have a

---

**Reviewer**	**3**
**Name**	**Lowres, Nicole**
**Affiliation**	**Heart Research Institute Ltd, Stroke Prevention**
**Date**	**25-Aug-2025**
**COI**	**None**

I have read this paper with interest and congratulate the authors on their work.

My following comments are made in an attempt to try and strengthen the manuscript.

1. Of the population that you selected participants from – how many were not eligible for participation and how many feel into each grouping, eg not able to use the watch, did not have a compatible smartphone – this is important to the qualitative data and wider implementation recommendations. These people that are ineligible are interesting and reportable results as they speak to the generalizability of the population. Out of 34 patients almost half were not eligible – so widespread uptake may not be great

2. The first paragraph of the results needs some clarification and I think a flow chart would help show the data nicely. From the way it reads: 34 patients were seen in the outpatient clinic – how many of these were not eligible and why; then 18 were included, but 11 of these declined (9 of them completed a non-participant questionnaire) so it seems like only 7 people completed the full interview.

3. I dont think you need to include the interview questions when you are quoting responses – just put in the relevant quote and put the P-number and details (as you have). I also don't think you need to label their quote with Participant or Interviewee as it is not standard practice and it is a given if you have the P-number. If you do keep this, then keep the descriptor consistent throughout the manuscript for all mentions of these people i.e. choose Participant or interviewee (convention uses participant)

4. Some of the quotes could be included in a sentence rather than as a stand alone quote. A good example is the quotes for contextual factors. The quote "not with the watch" is not very illustrative and could just be stated in a sentence. Maybe you have some other quotes to use instead, that could bring out some more nuances.

5. You mention Ding 2022 who used smartwatch PPG in stroke survivors, however there are a few other papers looking at patient experience of smart watch ECGs that you should compare your results too. Unfortunately it is not correct to state that you are the first study to look at this.

• Shih P, Prokopovich K, Degeling C, et al. Direct-to-consumer detection of atrial fibrillation in a smartwatch electrocardiogram: Medical overuse, medicalisation and the experience of consumers. Social Science & Medicine. 2022;

• Koshy AN, Ko J, Sajeev JK, et al. Evaluating patient attitudes and barriers towards smart technology for cardiac monitoring: results from a prospective multicentre study. BMJ Innovations. 2019

• A'Court C, Jenkins W, Reidy C and Papoutsi C. Patient-initiated cardiovascular monitoring with commercially available devices: How useful is it in a cardiology outpatient setting? Mixed methods, observational study. BMC Cardiovasc Disord. 2022

VERSION 1 - AUTHOR RESPONSE

Reviewer 1 – Dr. Carina Wennerholm:
Is the abstract accurate, balanced, and complete?
Comment 1: *It should be clear that a thematic analysis has been conducted.*
Response 1: We re-wrote the abstract and the following phrase to the design: *"Qualitative study using semi-structured interview and thematic analysis."*

Are the methods described sufficiently to allow the study to be repeated?
Comment 2: *Isn't this a prospective qualitative study with a deductive approach?*

Response 2: We agree with the reviewer. We altered the following sentences of the Method section: first sentence of paragraph study design: *"We conducted a prospective qualitative study in which […]"* And in the Interviews paragraph we now write: *"[…] using an interview guide with a deductive approach."*

Comment 3: *The theoretical framework of the Unified Theory of Acceptance and Use of Technology model, focusing on implementation outcomes? Deductive analysis? This needs to be made clear*
Response 3: As stated in response 2, we now explicitly write that we use a deductive approach. Furthermore, we re-wrote how the interview guide was developed because based upon the reviewer's comments this was not sufficiently clear: *"We developed a topic list based on the relevant implementation outcomes: acceptability, feasibility, appropriateness and sustainability as described by Peters et al. (10). […] To explain intentions to use and usage behaviour we used input from the Unified Theory of Acceptance and Use of Technology model (11). To explore social context we added aspects from the Social-Ecological Model (12). We tried to keep a broad scope in the interview guide to ensure a low threshold for participants to freely come up with additional themes or enriching information."*

Comment 4: *Missing a brief summary of the main content of the interview guide and then a reference, better than including the entire interview guide as an appendix.*
Response 4: We understand the reviewer's comment, however, since the development of the interview guide is already briefly discussed, results and presented themes are identical, and we are limited by the word count, we think adding this will not improve readability. We therefore decided not to add this to the main manuscript.

Comment 5: *Analysis: A more detailed description of the different steps of the analysis is desired.*
Response 5: We chose to limit the description of the conduction of our thematic analysis to limit our word count. Because of the reviewer's comment, and because we saw we were insufficiently clear about the method used, we altered the Analysis paragraph to the following: *"Interviews were transcribed verbatim and anonymized. Instead of transcript review, we conducted multiple "member checks" during interviews. We conducted a thematic analysis according to the six phases of Braun and Clarke (13). In the first twelve interviews, two researchers (EK and PV) independently carried out all phases, with discrepancies resolved in discussion with a third researcher (EMvC). For the last six interviews, one researcher (EK) performed the analysis, while the third researcher (EMvC) critically reviewed each step. The process was iterative: researchers familiarized themselves with the data, generated and compared initial codes in using MAXQDA Plus: 2022 (V.22.1.1) and then collaboratively developed, reviewed, and refined themes. Ultimately narratives were developed to define and name themes, and illustrative examples were added to enrich the final reporting. Inclusion ceased at saturation, as determined by all three analysts."*

Are research ethics (e.g., participant consent, ethics approval) addressed appropriately?
Comment 6: *Is there an ethics approval number?*
Response 6: We have added the waiver approval number in the Analysis paragraph.

Are the results presented clearly?
Comment 7: *As an overview and aid for the reader, it is desirable to have a table of the themes and sub-themes of the results at the very beginning of the results section.*
Response 7: Since we describe our results according to the coding tree, we added the table of the coding tree to the beginning of the results section: *"We describe our findings from the interviews according to the themes as described in the coding tree (Table 2)."*

Comment 8: *I think the first paragraph of the results belongs in the Methods section.*
Response 8: We believe the data presented here are a 'result' of our study selection methods. We therefore think it should be presented in the Results section. We did re-write this paragraph, see response 1 to reviewer 3.

Comment 9: *The thematic analysis seems a bit strange to me. Are the themes in the results abstracted? It seems based on the "topic list" in the interview guide, Acceptability and Perceived Appropriateness? How was the "topic list" developed? Based on the theoretical framework of the Unified Theory of Acceptance and Use of Technology model?*
Response 9: As also described in response 3, we agree that our previous description of the development of the topic list was insufficiently clear. We therefore re-wrote this section in the "Interviews" paragraph of the Method section. Due to our deductive approach and the intention to deepen participants' experiences and opinions regarding the described implementation outcomes, results were relatable to the used theoretical frameworks. Despite our best efforts our interviews did not result in new themes. For reasons of unambiguous terminology, we used the names of overarching themes as used as implementation outcomes.

Comment 10: *There also needs to be an explanatory text under each Theme.*
Response 10: In the Methods section's Interviews paragraph we added the following: *"We adhered to the definitions of these outcomes as described by Peters et al: acceptability, being defined as the perception among stakeholders that an intervention is agreeable; appropriateness as the fit or relevance of an intervention in a particular setting; feasibility as the extent to which an intervention can be carried out in a particular setting, and; sustainability as the extent to which an intervention is maintained or institutionalised in a given setting (10)."*

Reviewer 2 - Dr. Trudie Lobban:
Comment 1: *need to engage with patient organisations to reach a far larger group of patients rather than only 18. Organisations that specialise in heart rhythm disorders have a*
****editor's note: despite our best efforts, we were unable to obtain the full set of comments from this reviewer.*
Response 1: Unfortunately, part of the reviewer's comment has disappeared. Since our study has a qualitative research question and we reached data saturation, we are confident that results will not change significantly when a larger group of participants is included.
Notable to mention is that, in an effort to obtain funding, we did previously consult a patient collective (College of Experience-experts of the 'Harteraad'), which provided input for a qualitative

study with similar research question and setting (ambulatory 1L-ECG monitoring). In that study we planned to use the same patient selection method as used in the presented study. So this part of the presented study was in fact previously discussed with, and agreed by, a patient organisation.

Reviewer 3: - Dr. Nicole Lowres:
I have read this paper with interest and congratulate the authors on their work.
My following comments are made in an attempt to try and strengthen the manuscript.

Comment 1: *Of the population that you selected participants from – how many were not eligible for participation and how many feel into each grouping, eg not able to use the watch, did not have a compatible smartphone – this is important to the qualitative data and wider implementation recommendations. These people that are ineligible are interesting and reportable results as they speak to the generalizability of the population. Out of 34 patients almost half were not eligible – so widespread uptake may not be great*
Response 1: We thank the reviewer for this interesting suggestion. We agree this was insufficiently clear in the previous manuscript. Therefore we re-wrote the first paragraph to clarify these questions together with the comments as stated in comment 2. We now write: *"We invited 34 potentially eligible patients at the outpatient clinic for study-participation between January and August 2023 (see Figure 3 for flow-chart). Individuals ≥75 years had low presentation rates and often declined participation. Eleven patients declined participation, of which nine agreed to complete a non-participant questionnaire. These nine patients cited concerns about study complexity and smartphone compatibility – even prior to checking compatibility by our researchers (Supplementary Appendix III). Twenty-three patients were willing to participate, of which five were purposively excluded at the discretion of the investigators (PV and EK) based upon the purposive selection criteria. We ultimately included 18 patients (age: 32–85 years; 50% female, see Table 1). Mean DHLI scores ranged from 2.6 to 4.0, evenly distributed across categories ≥2.5–≤3.0, >3.0–≤3.5, and >3.5–4.0. Most participants (n=12) had no history of cardiovascular disease. Interviews lasted 22–55 minutes."*

Comment 2: *The first paragraph of the results needs some clarification and I think a flow chart would help show the data nicely. From the way it reads: 34 patients were seen in the outpatient clinic – how many of these were not eligible and why; then 18 were included, but 11 of these declined (9 of them completed a non-participant questionnaire) so it seems like only 7 people completed the full interview.*
Response 2: See response 1. We also added a flow-chart.

Comment 3: *I don't think you need to include the interview questions when you are quoting responses – just put in the relevant quote and put the P-number and details (as you have). I also don't think you need to label their quote with Participant or Interviewee as it is not standard practice and it is a given if you have the P-number. If you do keep this, then keep the descriptor consistent throughout the manuscript for all mentions of these people i.e. choose Participant or interviewee (convention uses participant)*

Response 3: We thank the reviewer for this suggestion. In all quotes but one, we removed the quotes of the interviewers. Here, we also removed the "Participant"-label.

Comment 4: *Some of the quotes could be included in a sentence rather than as a stand alone quote. A good example is the quotes for contextual factors. The quote "not with the watch" is not very illustrative and could just be stated in a sentence. Maybe you have some other quotes to use instead, that could bring out some more nuances.*
Response 4: We changed the quote for contextual factors to the following: *"I'm not easily embarrassed, but I can also imagine some people thinking: just forget about it [pressing the event button of the Holter monitor on the chest]… Especially as a woman, it's a bit less fun to pull up your shirt. With a watch, you don't have that problem, of course.* ***P16, male, 48 years***

Additionally, we agree with the reviewer that in some sections of the interview results, the description of our findings is sufficient and does not require the specific quote. We therefore deleted the quotes from the *Burden* and *Privacy* sections of the Results.

Comment 5: *You mention Ding 2022 who used smartwatch PPG in stroke survivors, however there are a few other papers looking at patient experience of smart watch ECGs that you should compare your results too. Unfortunately it is not correct to state that you are the first study to look at this.*
Response 5: We thank the reviewer for the critical and constructive feedback. Given that the suggested articles provide interesting depth to our discussion on our comparison with previous literature, we have added Shih et al and Koshy et al. to our discussion. In A'Court et al. no patient experiences are investigated, as this involved an evaluation among clinicians. As for your comment on originality, we agree that we are not the first to study stakeholders' experiences with smartwatch ECGs, however, we do believe that we are (to our knowledge) the first to offer a qualitative analysis of patient experiences with the use of a smartwatch 1L-ECG among primary care patients referred for rhythm monitoring in an ambulatory setting.
Because of the reviewers' comment we added the following to the 'comparison with existing literature' section of the discussion: *"Other studies investigating user experiences with a smartwatch 1L-ECG device have been done, however to our opinion these are difficult to compare to our study. Shih et al. interviewed early-consumers of the Apple watch with ECG feature. This was a relatively young population of 'worried-well' instigating self-screening with their Apple watch. Therefore this was an asymptomatic, relatively young population, using the 1L-ECG smartwatch device not initiated or supervised by a physician. The focus in their interviews, and thereby their results, were therefore not comparable to our setting and population. Koshy et al. also investigated patient experiences when simultaneously using a chest-worn device and smartwatch. However, these were hospitalized patients in a tertiary hospital, of whom almost half did not own a smartphone. Therefore, their population was not similar to an ambulatory primary care cohort with a requirement for smartphone ownership as in our study. Lastly, Koshy et al. used a questionnaire instead of a qualitative method to further explore patients' experiences and views."*

VERSION 2 - REVIEW

**Reviewer**	**3**
**Name**	**Lowres, Nicole**
**Affiliation**	**Heart Research Institute Ltd, Stroke Prevention**
**Date**	**17-Oct-2025**

COI

Thank you for the opportunity to review the revised article. I appreciate the authors attempts to address each of the reviewer comments. I feel that the manuscript has improved, but there are still many comments where the responses need further work.

1. I am still a bit confused about how many people were interviewed in the end? Were 18 people interviewed? Maybe this needs to be stated somewhere in your flow chart and description. I would look at some other papers to see how they present the information in flow-charts, and revise the flowchart accordingly. I would also add the information to the flow chart for the 5 people that you purposively excluded and why each one was excluded. You don't need to present all the information in the text if it is clear in the flowchart.

2. The results could use some revising and more detail. If 18 people were interviewed, then you should have quite a lot of quotes to use. There are only mentions of 7 different participants in your results which is a concern as you interviewed 18.

I would revise the results to provide more information and more quotes. To give some depth to the results you could actually state the number of people that reported that idea (E.g. instead of saying "some people" you could say "12 people agreed that ...." or you could give all the P numbers for the people that agreed with that statement).

3. Make sure you only include relevant bits of the quote. Streamline the excess words out so the reader is clear about what they should take from the quote

Example, this quote:

No. No. But I have to say as well: look, I find it easy. You explained that you should actually do it like this. But at the moment when you're not feeling well, with the heart, especially at night, then you're not really - I certainly am not - able to think: oh, I should sit like this first. P5, female, 58 years

Could be something like this:

"I find it easy... But ... when you're not feeling well, with the heart, especially at night, then you're not really ... able to think: oh, I should sit like this first." P5, female, 58 years

4. Figure 2 also needs more detail, it is currently not really themes as much as key words

5. There is a lot of repetition through the methods section that could be cut to make the paper more readable and to save some word count for adding value to the results section. I don't think that English is the native language of the authors, so this may make it hard to write in a clear and succinct way. I would suggest that you ask a native English speaker to assist with structuring the paper (particularly the methods) so that the sentences are clear and you only say something once in the paper. For example, some of the data in the study design section actually belongs in the participant selection section.

6. The ethics statement etc should be placed in the study design section, not at the end of the analysis section

---

VERSION 2 - AUTHOR RESPONSE

Reviewer 3: - Dr. Nicole Lowres:

Thank you for the opportunity to review the revised article. I appreciate the authors attempts to address each of the reviewer comments. I feel that the manuscript has improved, but there are still many comments where the responses need further work.

Comment 1: *I am still a bit confused about how many people were interviewed in the end? Were 18 people interviewed? Maybe this needs to be stated somewhere in your flow chart and description. I would look at some other papers to see how they present the information in flow-charts, and revise the flowchart accordingly. I would also add the information to the flow chart for the 5 people that you purposively excluded and why each one was excluded. You don't need to present all the information in the text if it is clear in the flowchart.*

Response 1: We thank the reviewer for this helpful suggestion. We have clarified the total number of interviewees (n = 18) in both the Participants section and the flow-chart in Figure 3. For each of the excluded cases, there was not one particular reason which we can state in the flowchart as 'reason for exclusion'. Rather, is was due to a combination of sex, age and DHLI, as stated in the patient selection paragraph of the method section: *"Twenty-three patients were willing to participate, of which five were purposively excluded due to (a combination of) being woman, being of younger age, or having higher DHLI, as such participants were already sufficiently represented in our sample (see baseline characteristics of the n=5 excluded patients in the flow-chart)."* The flowchart has therefore been revised accordingly to present the full inclusion process more transparently, including the baseline characteristics of the five individuals who were purposively excluded.

We agree that some information was presented twice. Therefore, we removed overlapping details from the text that are already shown in the flow-chart or in Table 1.

Comment 2: *The results could use some revising and more detail. If 18 people were interviewed, then you should have quite a lot of quotes to use. There are only mentions of 7 different participants in your results which is a concern as you interviewed 18.*

I would revise the results to provide more information and more quotes. To give some depth to the results you could actually state the number of people that reported that idea (E.g. instead of saying "some people" you could say "12 people agreed that ……." or you could give all the P numbers for the people that agreed with that statement).

Response 2: We thank the reviewer for this valuable suggestion. We agree that the number of quotes included in the previous version was too limited. We have therefore added several additional illustrative quotes throughout the Results section (see text), to ensure that the findings better reflect the breadth of perspectives shared by the 18 participants.

With regard to the suggestion to quantify the number of participants expressing specific views, we carefully considered this but decided not to include numerical counts. Our study employed a qualitative, purposive sampling approach designed to capture a broad diversity of perspectives rather than to establish prevalence or frequency. In this context, reporting the number of participants who “agreed” or “disagreed” might inadvertently suggest a quantitative interpretation of qualitative data. Moreover, not all participants were asked identical questions in the same way, and individual responses often reflected nuance rather than discrete agreement or disagreement. We therefore consider a qualitative presentation of patterns and themes—supported by a wider range of participant quotes—the most appropriate way to represent our findings.

Comment 3: *Make sure you only include relevant bits of the quote. Streamline the excess words out so the reader is clear about what they should take from the quote*
Example, this quote:
No. No. But I have to say as well: look, I find it easy. You explained that you should actually do it like this. But at the moment when you're not feeling well, with the heart, especially at night, then you're not really - I certainly am not - able to think: oh, I should sit like this first. P5, female, 58 years
Could be something like this:
“I find it easy… But … when you're not feeling well, with the heart, especially at night, then you're not really … able to think: oh, I should sit like this first.” P5, female, 58 years
Response 3: We agree with the reviewer that some quotes were transcribed too literally, which made them less clear. In response, we have carefully reviewed and edited all quotes in the manuscript to ensure that only the most relevant and meaningful excerpts are included, and that they are presented in a clearer and more concise manner.

Comment 4: *Figure 2 also needs more detail, it is currently not really themes as much as key words*
Response 4: We thank the reviewer for this comment. We believe the reviewer was referring to Table 2. To clarify the hierarchical structure of our thematic analysis, we have revised Table 2 by adding column headers (‘Theme’, ‘Sub-theme’, ‘Subcategory’) and an explanatory note in the legend. This should make clear that the table summarises the structure of themes and sub-themes rather than listing keywords.

Comment 5: *There is a lot of repetition through the methods section that could be cut to make the paper more readable and to save some word count for adding value to the results section. I don’t think that English is the native language of the authors, so this may make it hard to write in a clear and succinct way. I would suggest that you ask a native English speaker to assist with structuring the paper (particularly the methods) so that the sentences are clear and you only say something once in the paper. For example, some of the data in the study design section actually belongs in the participant selection section.*
Response 5: We agree with the reviewer that limiting the word count in the Methods section would improve readability. However, due to the dual study design - comprising both an interventional component (simultaneous use of smartwatch and Holter) and a qualitative component - it is important that readers clearly understand both the context in which patient experiences were gathered and the methodological framework used for the qualitative part (i.e. the SRQR checklist items). These factors make it challenging to substantially shorten this section without losing

important detail. Nevertheless, we have revised the text to make it more concise, structured and clear. Furthermore, we consulted colleagues with relevant English language expertise.

Comment 6: *The ethics statement etc should be placed in the study design section, not at the end of the analysis section.*
Response 6: The ethics statement has been moved from the end of the analysis section to the study design paragraph.

VERSION 3 - REVIEW

**Reviewer**	**3**
**Name**	**Lowres, Nicole**
**Affiliation**	**Heart Research Institute Ltd, Stroke Prevention**
**Date**	**16-Nov-2025**
**COI**	

The authors have considered the feedback and revised the article nicely.

There is one remaining suggestion that I would make, which parallels a suggestion I made in the first round of feedback. The work would look a lot more professional if the quotes do not include the "interviewer question" followed by the response.

The remaining example of this in the manuscript is:

One participant (P8), contacted the out-of-hours GP service upon receiving this notification, while the other (P1) experienced anxiety but followed our instructions to act on the physical complaints only.

Interviewer: And what did that cause for you [notification of irregular heartbeat with advice to contact a healthcare provider]? Did it make you anxious or scared? […]

Participant: To some extent, yes, but as I said before: I just know what causes it. It's just due to stress. Then the heart races. I don't worry extra about that. […]

Interviewer: Did you hesitate to contact the healthcare provider?

Participant: Uhm, yes, of course, you don't just, things really need to go wrong before you actually contact your healthcare provider P1, male, 71 years

This would be better solely written as a quote - which can be achieved using something like the following:

One participant (P8), contacted the out-of-hours GP service upon receiving this notification. However, the other (P1) experienced anxiety but followed our instructions to act on the physical complaints only.

[I felt anxiety] to some extent, but … I just know what causes it. It's just due to stress. Then the heart races. I don't worry extra about that. … Yes, of course [I hesitated to call the healthcare provider] … things really need to go wrong before you actually contact your healthcare provider P1, male, 71 years
